# Supplementary material for: Analysis of Melanoma Gene Expression Signatures at the Single-Cell Level Uncovers 45-Gene Signature Related to Prognosis
Source: Biomedicines. 2022 Jun 22;10(7):1478. doi: 10.3390/biomedicines10071478 (PMC9313451; doi:10.3390/biomedicines10071478)
Supplement: Supplementary file 1 [file biomedicines-10-01478-s001.zip › biomedicines-1753526-supplementary figures.pdf]

Supplementary Figures:

Figure S1

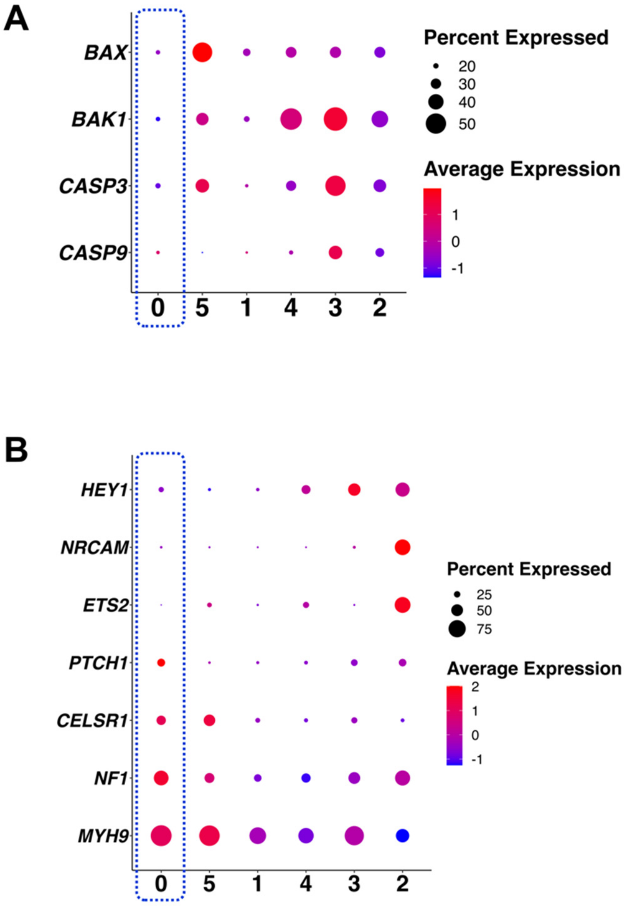

Figure S1: Anti-apoptotic characteristics of cluster 0.

(**A,B**) Dot plots illustrate the expression levels of (**A**) the pro-apoptotic genes (*BAX*, *BAK1*, *CASP3*, and *CASP9*), and (**B**) Hedgehog (Hh) signaling pathway-related genes (*MYH9*, *NF1*, *CELSR1*, *PTCH1*, *ETS2*, *NRCAM*, and *HEY1*) across the melanoma cell clusters.

Figure S2

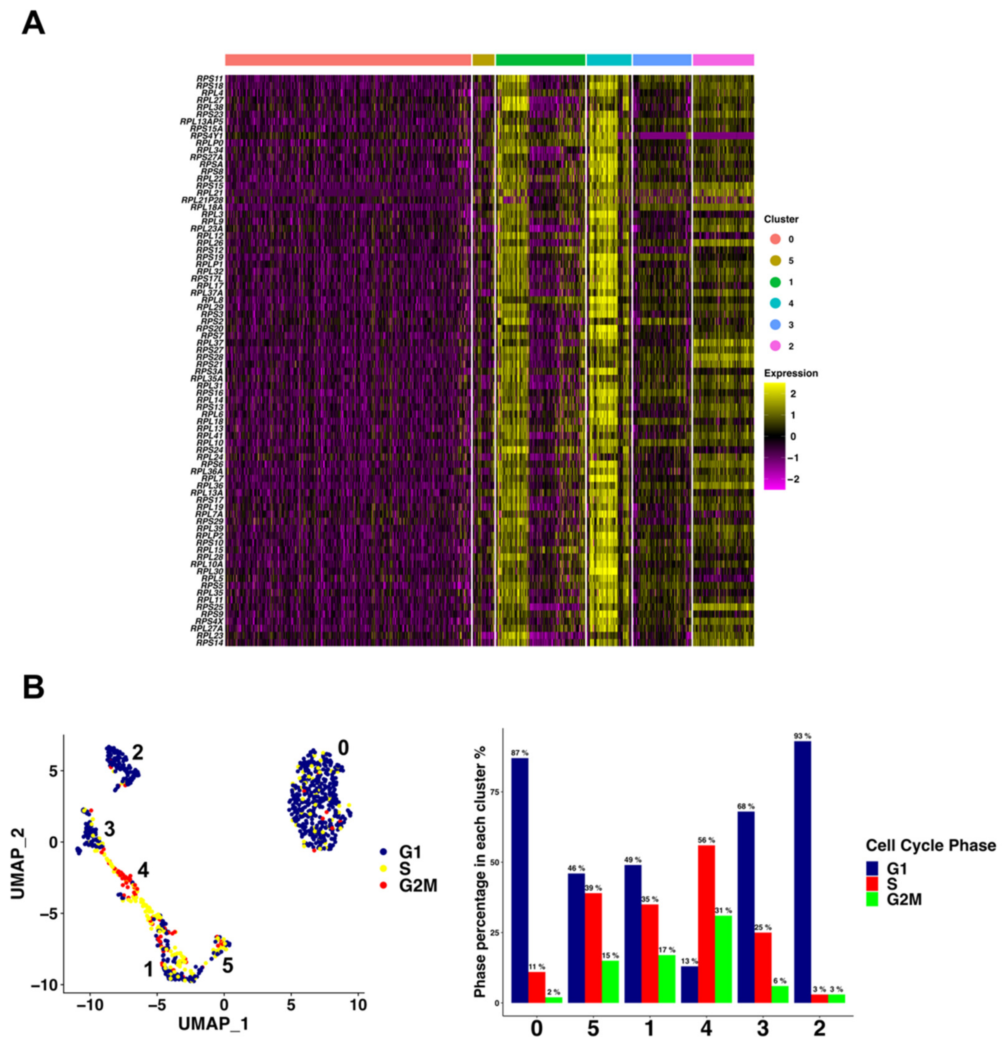

Figure S2: Ribosomal and cell cycle characterization of the melanoma cell clusters. (**A**) Expression levels of ribosomal proteins in melanoma cell clusters visualized by a heatmap. (**B**) Cell cycle phases (G1, S, and G2M) across the melanoma cell clusters are visualized by the UMAP plot (left), and the percentages of cell cycle phases in each cluster (right bar plot).

Figure S3

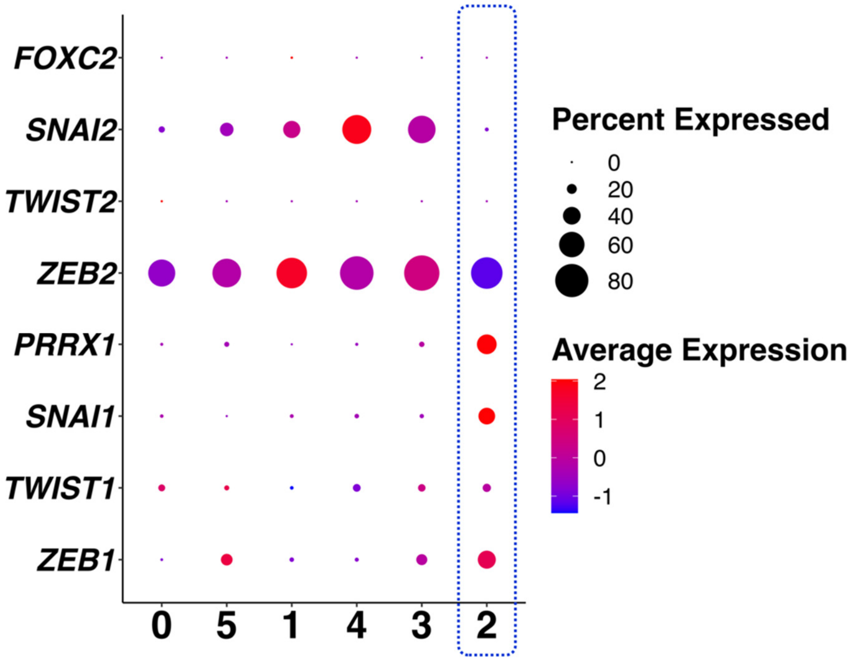

Figure S3: EMT-TFs highly expressed in cluster 2.

Dot plot showing the expression levels of the EMT-TFs (*ZEB1/2*, *TWIST1/2*, *SNAI1/2*, *PRRX1*, and *FOXC2*) across the melanoma cell clusters. Dotted line rectangle indicates the highest expression levels of the EMT-TFs in cluster 2.

Figure S4

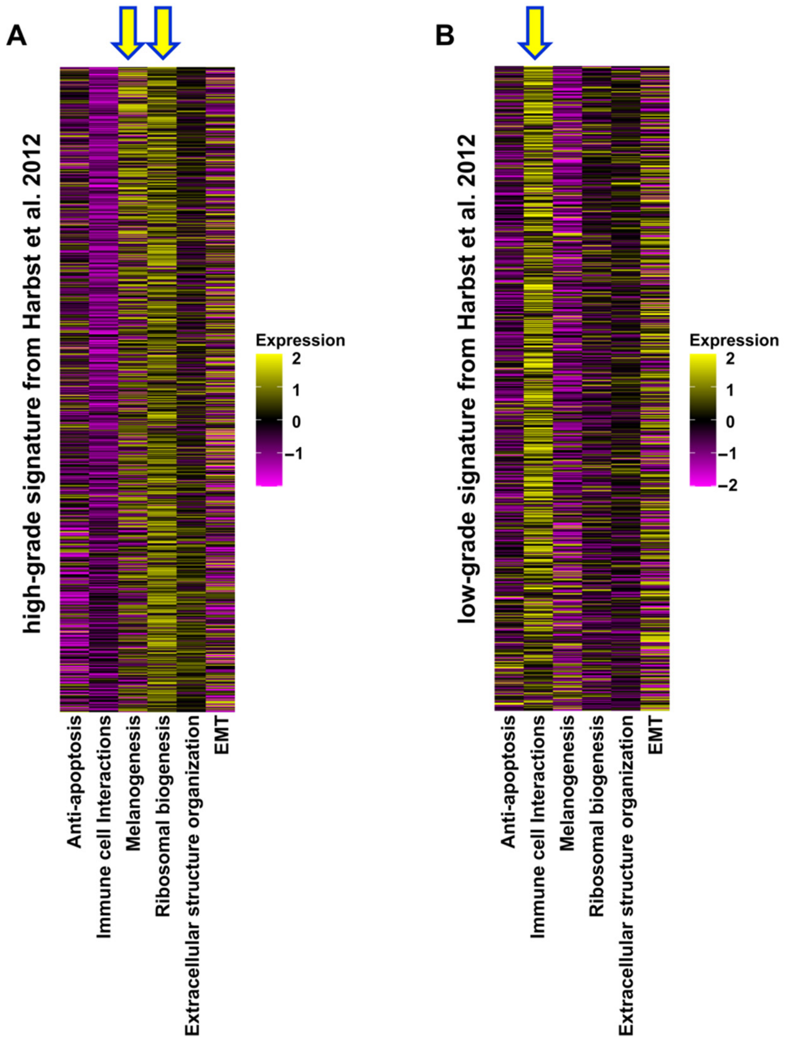

Figure S4: The identified GESs partially overlapped with "high-grade" and "low-grade" GESs.

(**A,B**) Heatmaps show the expression of the representative genes of (**A**) "high-grade" GES (left heatmap) and (**B**) "low-grade" (right heatmap) GESs in our identified TCGA GESs. Arrows indicate GESs with the highest similarity.

Figure S5

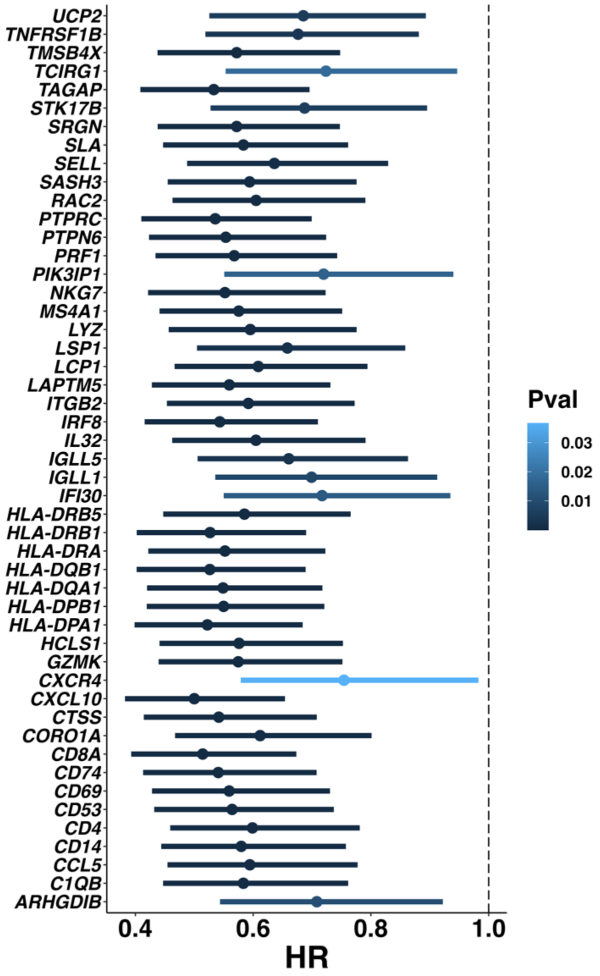

Figure S5: Univariate Cox regression analyses for DEGs of "Immune cell interactions" GES.

Forest plot for univariate Cox regression analyses of 49 out of 56 DEGs (|Log2FC| > 1.5, $p < 0.05$) that were significantly correlated with prognosis ($p < 0.05$). All 49 DEGs were associated with improved survival ($p < 0.05$ and HR < 1).
